# Long-Term Fructose Intake Increases Adipogenic Potential: Evidence of Direct Effects of Fructose on Adipocyte Precursor Cells

**DOI:** 10.3390/nu8040198

**Published:** 2016-04-02

**Authors:** María Guillermina Zubiría, Ana Alzamendi, Griselda Moreno, María Amanda Rey, Eduardo Spinedi, Andrés Giovambattista

**Affiliations:** 1 Neuroendocrinology Laboratory, Multidisciplinary Institute of Cellular Biology (IMBICE, CICPBA-CONICET-UNLP), Calles 526 10 y 11, La Plata 1900, Argentina; gzubiria@imbice.gov.ar (M.G.Z.); aalzamendi@imbice.gov.ar (A.A.); mamandarey@gmail.com (M.A.R.); 2 Biology Department, School of Exact Sciences, Universidad Nacional de La Plata, La Plata 1900, Argentina; 3 Institute of Immunological and Physiopathological Research (IIFP, CONICET-UNLP), School of Exact Sciences, Universidad Nacional de La Plata, La Plata 1900, Argentina; gmoreno@iifp.laplata-conicet.gov.ar; 4 Center of Experimental and Applied Endocrinology (CENEXA, UNLP-CONICET, PAHO/WHO Collaborating Center for Diabetes), La Plata Medical School, Universidad Nacional de La Plata, La Plata 1900, Argentina; spinedi@cenexa.org

**Keywords:** retroperitoneal adipose tissue, adipogenesis, SVF cells, precursor cell competency

## Abstract

We have previously addressed that fructose rich diet (FRD) intake for three weeks increases the adipogenic potential of stromal vascular fraction cells from the retroperitoneal adipose tissue (RPAT). We have now evaluated the effect of prolonged FRD intake (eight weeks) on metabolic parameters, number of adipocyte precursor cells (APCs) and *in vitro* adipogenic potential from control (CTR) and FRD adult male rats. Additionally, we have examined the direct fructose effects on the adipogenic capacity of normal APCs. FRD fed rats had increased plasma levels of insulin, triglyceride and leptin, and RPAT mass and adipocyte size. FACS studies showed higher APCs number and adipogenic potential in FRD RPAT pads; data is supported by high mRNA levels of competency markers: PPAR*γ*2 and Zfp423. Complementary *in vitro* experiments indicate that fructose-exposed normal APCs displayed an overall increased adipogenic capacity. We conclude that the RPAT mass expansion observed in eight week-FRD fed rats depends on combined accelerated adipogenesis and adipocyte hypertrophy, partially due to a direct effect of fructose on APCs.

## 1. Introduction

In the last few decades, obesity, metabolic syndrome and type 2 diabetes mellitus (DMT2) have become serious problems for health systems, emerging as a worldwide epidemic. These metabolic disorders are multifactorial depending on genetic background as well as environmental and behavioral factors, such as eating habits. The quantity and quality of modern diets, in particular the increase of sugar intake, have been associated with the high incidence of metabolic disorders [1,2]. In fact, high fructose consumption, mainly through sweetened drinks, has been linked to deleterious metabolic consequences, such as insulin resistance, dyslipidemias, increased abdominal adipose tissue (AAT) mass and changes in the pattern of AT adipokine secretion [3,4].

The deleterious metabolic effects of high fructose intake have been related to the activating role in the *de novo* lipogenesis pathway in the liver, and therefore stimulating AT fatty acid uptake. This stimulation of *de novo* lipogenesis is not so markedly increased by other carbohydrates [5], mainly because fructose bypasses the main control point of glycolysis, the phosphofructokinase step, and is converted into trioses [6], precursors of triglyceride synthesis. Although most of the absorbed fructose is extracted and metabolized by the liver [7], a small amount remains in circulation and can be absorbed through its specific cellular transporter GLUT-5 by tissues such as kidney and AT [8,9].

The increase in AAT mass has been considered one of the main disorders associated with fructose overconsumption. AT expansion is the result of two independent processes: hyperplasia and hypertrophy. Dysfunctional AT is correlated with adipocyte size rather than AT mass. In fact, hypertrophic adipocytes are insulin resistant; secrete more leptin and pro-inflammatory cytokines and less adiponectin [10,11,12]. AT hyperplasia is the consequence of activated adipogenesis; a process involving two stages: the determination of the adipocyte precursor cells (APCs) and terminal cell differentiation, wherein APCs change into adipocytes. The ability of APCs to turn into adipocytes after specific stimulation is known as APCs competency and it is a characteristic of determined cells [13].

As we have previously shown, fructose rich diet (10% w/v, FRD) intake induces several metabolic alterations related to AT mass expansion [14,15,16]; however the activation or inhibition of adipogenesis in this phenomenon remains unclear. In the 3T3L1 preadipocyte cell line, the addition of fructose to the culture medium stimulates the terminal stage of adipogenesis, a mechanism that is dependent on the specific fructose transporter, GLUT-5 [17,18]. We earlier demonstrated that adult rats fed with FRD for three weeks showed APCs from retroperitoneal AT (RPAT) displaying high levels of competency markers, such as PPARγ2 and Zfp423 [15]. Nevertheless, the *in vivo* impact of FRD intake for a longer period of time and the direct *in vitro* effect of fructose on APCs adipogenic potential and number have not been studied up to now. Our study provides, for the first time, evidence that high fructose intake for eight weeks increases, probably through a direct effect, the number and competency of APCs from RPAT, therefore favoring adipocyte differentiation and contributing to its mass expansion.

## 2. Material and Methods

### 2.1. Animals and Treatment

Normal adult male Sprague-Dawley rats (60 days of age) were kept in a temperature-controlled environment (20–22 °C and fixed 12 h light/12 h dark cycle, lights on at 07:00 a.m. and fed *ad libitum* with Purina commercial rat chow. Rats were divided into two groups: one was provided with tap water only (CTR) and, the other, a solution of 10% fructose (w/v, Sigma-Aldrich, St. Louis, MO, USA) in tap water for eight weeks (conventionally called fructose rich diet, FRD). Rats were euthanized under non-fasting conditions (between 08:00 a.m. and 09:00 a.m.) and trunk blood was collected; plasma samples were then frozen (−20 °C) until metabolite measurements were taken. RPAT was aseptically dissected (brown adipose tissue vessels surrounding the kidney were removed and discarded), weighed and kept in sterile Dulbecco’s Modified Eagle’s Medium-Low Glucose (1 g/L) (DMEM-LG) for further procedures. Animals were euthanized according to protocols for animal use, in agreement with NIH guidelines for the care and use of experimental animals. All experiments were approved by our Institutional Animal Care Committee.

### 2.2. Peripheral Metabolite Measurements

Plasma levels of leptin (LEP), insulin (INS) and corticosterone (CORT) were determined by specific radioimmunoassays (RIAs) previously developed in our laboratory [19]. Circulating glucose (Glu, Wiener Argentina Lab., Rosario, Argentina) and triacylglycerols (Tg, Wiener, Rosario, Argentina) levels were measured using commercial kits.

### 2.3. RPAT Stromal Vascular Fraction (SVF) Cell and Adipocyte Isolation

Fresh RPAT pads were dissected, weighed and digested with collagenase as previously reported [20]. Briefly, fat tissue was minced and digested using 1 mg/mL collagenase solution in DMEM (at 37 °C, for 1 h). After centrifugation (1000 rpm, during 15 min), floating mature adipocytes were separated and reserved for later measurement. SVF pellets were filtered (in a 50 μm mesh nylon cloth) and washed with DMEM-LG (×3). SVF cells, containing APCs, were then resuspended in DMEM-LG supplemented with 10% (v/v) fetal bovine serum (FBS), HEPES (20 nM), 100 IU/mL penicillin and 100 μg/mL streptomycin (basal medium).

### 2.4. Adipocyte Size Analysis

The size of the isolated fat cells was measured as previously described [21], with minor changes. Briefly, a 50–150 µL aliquot from the top layer (floating cells) was added to 450 µL DMEM. 5–10 µL from the cell suspension were placed into a Neubauer chamber and cover-slipped. Five representative pictures from each sample were taken using a Nikon Eclipse 50i microscope equipped with a camera (Nikon Digital Sight D5-U3, Melville, NY, USA). Cell diameter was measured with image analysis software (Image ProPlus6.0, Rockville, MD, USA). Values below 25 µm were discarded as they can be considered lipid droplets. Values were recorded and assigned to groups differing by 10 µm diameter, creating a histogram with 10 µm-bins. Histograms were used to determine whether the distribution of adipocyte diameters was either normal or binomial, and to assess the presence of different sized, adipocyte sub-populations. We measured an average of 500–600 cells per field to calculate average adipocyte size.

### 2.5. RPAT SVF Cell Culture

RPAT SVF cells from CTR and FRD groups were seeded (2 × 10^4^ cells/cm^2^) in 24-well plates (Greiner Bio-One, Kremsmünster, Austria) and cultured in the basal medium at 37 °C in a 5% CO_2_-atmosphere [20]. Cells were induced to differentiate and processed for several determinations as described in more detail below (Materials and Methods section: 2.8; 2.9; 2.10; 2.11). Additionally, normal SVF cells from RPAT from control adult male rats were seeded and cultured for 7 days in the basal medium alone (Basal) or supplemented with 1 g/L of fructose (FRU) or 1 g/L glucose (GLU). After this period cells were immediately processed for RNA extraction or FACS analysis, or differentiated to adipocytes for lipid content determination, as described below.

### 2.6. SVF Cell Composition Analysis by Flow Cytometry (FACS)

Freshly isolated or cultured SVF cells from RPAT pads (at least 2 × 10^5^ cells in 100 µL PBS/0.5% BSA) were incubated with fluorescent antibodies or respective isotype controls for 1 h at 4 °C. After washing steps, flow cytometry was performed using a FACSCalibur flow cytometer (Becton Dickinson Biosciences, San Jose, CA, USA). A combination of cell surface markers were used to identify APCs as: CD34^+^/CD45^−^/CD31^−^ for freshly isolated SVF cells and CD34^+^/CD31^−^ for cultured SVF cells [22]. Conjugated monoclonal antibodies used were: anti-rat CD34:PE, anti-rat CD45:FITC, and anti-rat CD31:FITC (1 µg/1 × 10^6^ cells, Santa Cruz Biotechnology Inc., Santa Cruz, CA, USA). Samples were analyzed using CellQuest Pro (Becton-Dickinson, San Jose, CA, USA) and FlowJo softwares (TreeStar, San Carlo, CA, USA).

### 2.7. Cell Differentiation

Proliferating SVF cells (having reached 70%–80% confluence after 5–6 days of culture) were induced to differentiate by the addition of a differentiation mix containing 5 µg/mL insulin, 0.25 μM DXM, 0.5 mM 3-isobutyl-L-methylxanthine (IBMX) in the basal medium [20]. After 48 h media were removed and replaced with the fresh basal medium containing 5 µg/mL insulin. Cell samples were harvested after 10 days of the induction of differentiation (Dd10) and processed for several determinations, as described below. Medium samples were taken on Dd10 and kept frozen at −20 °C until measurement of leptin concentrations (see below).

### 2.8. RNA Isolation and Quantitative Real-Time PCR (qRT-PCR)

Total RNA was isolated from cells by the Trizol extraction method (Invitrogen, Life Tech., Carlsbad, CA, USA). Total RNA was reverse-transcribed using random primers (250 ng) and RevertAid Reverse Transcriptase (200 U/μL, Thermo Scientific, Vilnius, Lithuania). Two μL cDNA were amplified with HOT FIRE Pol EvaGreenqPCR Mix Plus (Solis BioDyne, Tartu, Estonia) containing 0.5 μM of each specific primer, using LightCycler Detection System (MJ Mini Opticon, Biorad, CA, USA). PCR efficiency was near 1. Expression levels were analyzed for β-actin (ACTβ, reporter gene), Adiponectin (ADIPOQ), CCAAT/enhancer binding protein alpha (C/EBPα), glucose transporter (GLUT-4), fructose transporter (GLUT-5) glucocorticoid receptor (GR), insulin receptor 1 (IRS-1) and 2 (IRS-2), Leptin (Ob), mineralocorticoid receptor (MR), Peroxisome Proliferator-Activated Receptor gamma 2 (PPAR-γ2), Preadipocyte Factor 1 (Pref-1), wingless-type MMTV integration site family member 10b (WNT-10b) and Zinc finger protein 423 (Zfp423). Designed primers are shown in alphabetical order in Table 1. Relative changes in the expression level of one specific gene (ΔΔCt) were calculated by the ΔCt method.

### 2.9. Leptin Measurement

Medium leptin concentration was determined by specific RIA [23]. In this assay, the standard curve ranged between 50 and 12,500 pg/mL, coefficients of variation intra- and inter-assay of 4%–7% and 9%–11%, respectively.

### 2.10. Cellular Lipid Content

On Dd 10, cells were washed with PBS and fixed with 10% formalin (for 10–15 min) in PBS. Then cells were quickly washed with PBS and stained for 1 h with Oil-Red O (ORO) solution (2:3 v/v H_2_O:isopropanol, containing 0.5% ORO) [24]. After staining, cells were washed (×3 with PBS) and dye from lipid droplets was extracted by adding isopropanol (10 min). To quantify cell lipid content, sample OD was obtained at 510 nm in a spectrophotometer. Remaining cells were digested with 0.25% Trypsin solution in PBS-EDTA, at 37 °C for 24 h and centrifuged at 8000× *g* for 15 s. OD of supernatants was read at 260 nm for DNA quantification and cell lipid content (measured by ORO and expressed in OD units) was then expressed by the corresponding cell DNA content.

### 2.11. Percentages of Cell Differentiation and Maturation

On Dd 10, differentiated cells were fixed with 10% formalin solution for 1 h at room temperature, and then stained using the Papanicolaou technique. The percentage of differentiated cells was calculated by counting the total number of cells and that of cells containing lipid droplets, when visualized in a light microscope (after counting 200–250 total cells per layer, at 40× magnification). Lipid-containing cells were assigned to one of the 3 graded stages of maturation according to the nucleus position: stage I (central), stage II (between central and peripheral), and stage III (completely peripheral) [25]. Percentages of cells corresponding to each maturation stage were expressed in relation to the total number of differentiated cells. Image analysis was assessed using a light Nikon Microscope and image analysis software (Image ProPlus6.0, Rockville, MD, USA).

### 2.12. Statistical Analysis

Results are expressed as mean values ± SEM. Data were analyzed by ANOVA (one-way) followed by Fisher’s test. To determine the differential effect of the treatment according to age, ANOVA (two-ways) was performed followed by Bonferroni’s test. The non-parametric Mann-Whitney test was used to compare adipocyte size populations between groups. Normal or binomial distribution of adipocyte size data was determined by Kruskall-Wallis test, followed by Mann-Whitney test. *P* values lower than 0.05 were considered statistically significant. All statistical tests were performed using GraphPad Prism 6.0.

## 3. Results

### 3.1. Effect of FRD Intake on Metabolic Parameters

**Figure 1:**
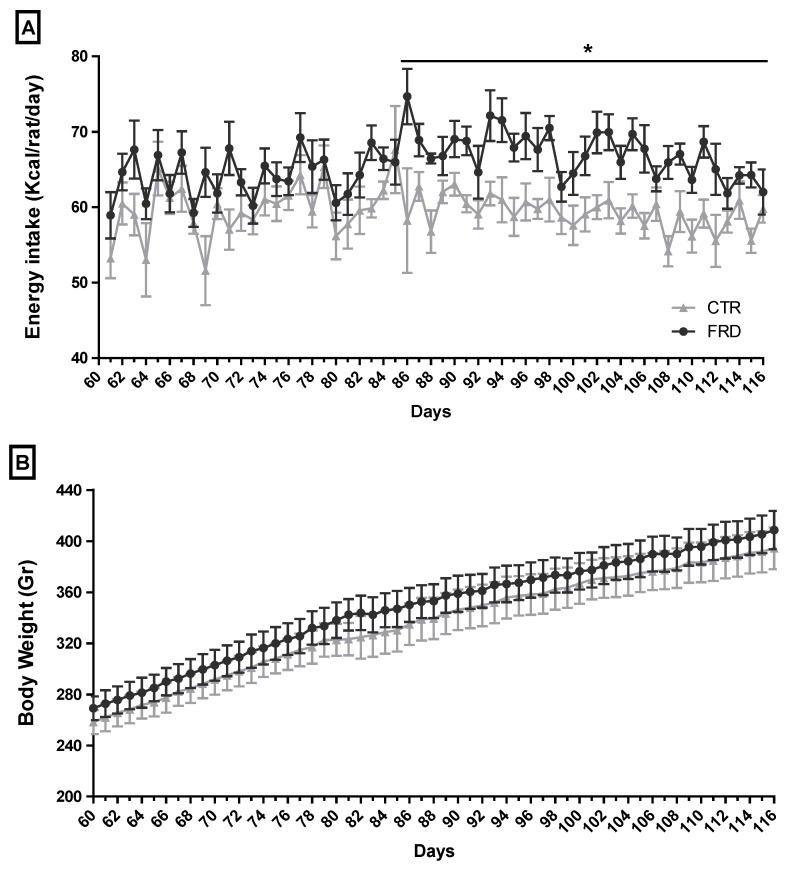
Food-derived energy intake and body weight of CTR and FRD rats. (**A**) Caloric intake and (**B**) body weight of CTR and FRD rats. Values are means ± SEM (*n* = 8 rats per group). * *p* < 0.05 *vs*. CTR values on the same day.

**Figure 2:**
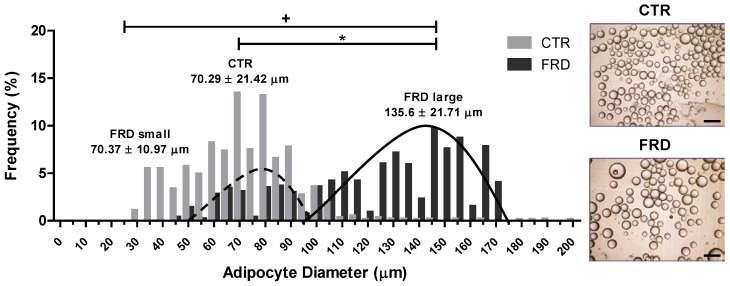
RPAT adipocyte diameter distribution. Dotted and continuous lines represent FRD small and large populations of adipocytes, respectively. Values are means ± SEM (*n* = 3 animals per group). Representative images of CTR and FRD mature adipocytes in cell suspension are shown (right, magnification 10×). Scale bars at 200 μm.

FRD rats showed high average of total energy intake from three up to eight weeks of treatment (Figure 1A), although they did not exhibit differences in body weight (Figure 1B). However, FRD intake significantly increased INS, Tg and LEP plasma levels, and also induced a significant increase in RPAT depot (Table 2). When we determined the size distribution of mature adipocytes contained in the RPAT we found two adipocyte populations, one similar to and another larger than CTR adipocyte population (Figure 2). The presence of small adipocytes in FRD rats may suggest that the generation of new adipocytes took place (adipogenesis), whereas large adipocytes could be the result of enlargement of preexisting fat cells, leading to adipocyte hypertrophy.

### 3.2. FRD Modifies APCs Number and Adipogenic Potential

**Figure 3:**
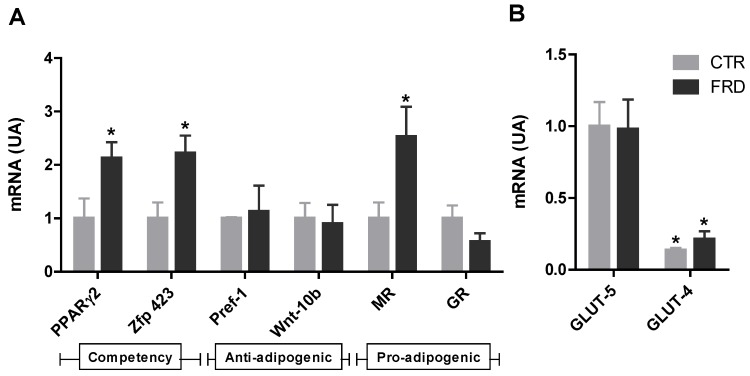

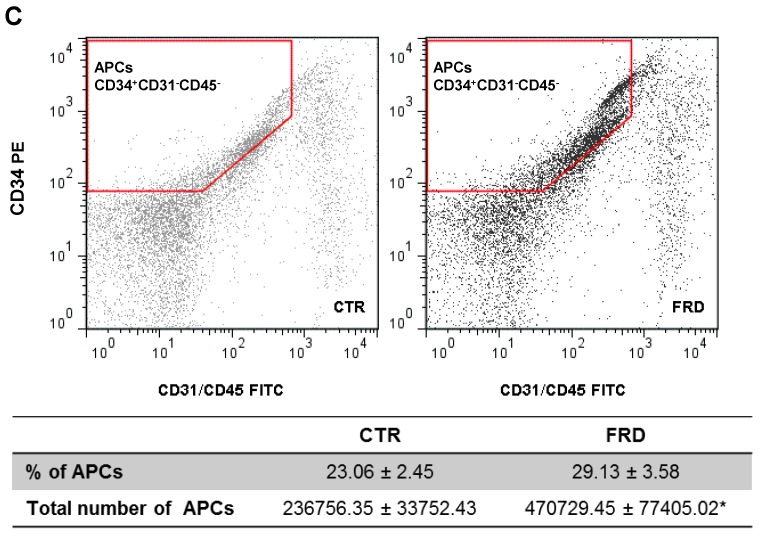
Effect of FRD intake on the APCs adipogenic potential and number. (**A**) Gene expression levels of cell competency (PPAR-γ2 and Zfp423), anti-adipogenic (Pref-1 and Wnt-10b) and pro-adipogenic (GR and MR) markers. * *p* < 0.05 *vs*. CTR values; (**B**) Gene expression levels of the specific fructose (GLUT-5) and glucose (GLUT-4) transporters in SVF cells in RPAT from CTR and FRD rats. Relative values to GLUT-5 expression in CTR cells. (AU: arbitrary units). Values are means ± SEM (*n* = 4 different experiments). * *p* < 0.05 *vs*. GLUT-5 values from each group; (**C**) APCs number in SVF in RPAT from CTR and FRD rats. APCs were identified by FACS analysis using CD34^+^CD45^−^CD31^−^ profile (indicated in red borders). Fluorescence profiles obtained for IgG isotype controls are shown in Figure S1 (Supplementary materials) FITC: fluorescein isothiocyanate; PE: phycoerythrin. Values are means ± SEM (*n* = 3/4 different experiments).

To test the effect of FRD intake on the adipogenic capacity of APCs, we measured the mRNA levels of anti-adipogenic, pro-adipogenic and competency factors expressed by these cells contained in the SVF. Freshly isolated SVF cells from FRD pads expressed significantly (*p* < 0.05 *vs*. CTR cells) higher levels of Zfp423 and PPARγ2 (competency markers), and the pro-adipogenic factor MR (Figure 3A). Conversely, no differences were found in Pref-1 and Wnt-10b mRNA levels (anti-adipogenic factors, Figure 3A).

We also evaluated the expression levels of the specific fructose transporter GLUT-5 in APCs. We found that this gene was expressed in APCs, although no differences were noticed in either group of cells; the same phenomenon occurred when GLUT-4 mRNA expression was quantified. Importantly, the APCs expression levels of GLUT-4 were markedly lower than those of GLUT-5, regardless of the cell-group examined (Figure 3B).

Interestingly, the number of APCs contained in the SVF of RPAT pads, was significantly higher in the FRD rats (Figure 3C). Collectively, these results strongly reveal that high fructose intake affects the adipogenic potential of APCs, changing their ability to respond to the differentiation stimuli and their cell number.

### 3.3. In vitro Adipocyte Differentiation

**Figure 4:**
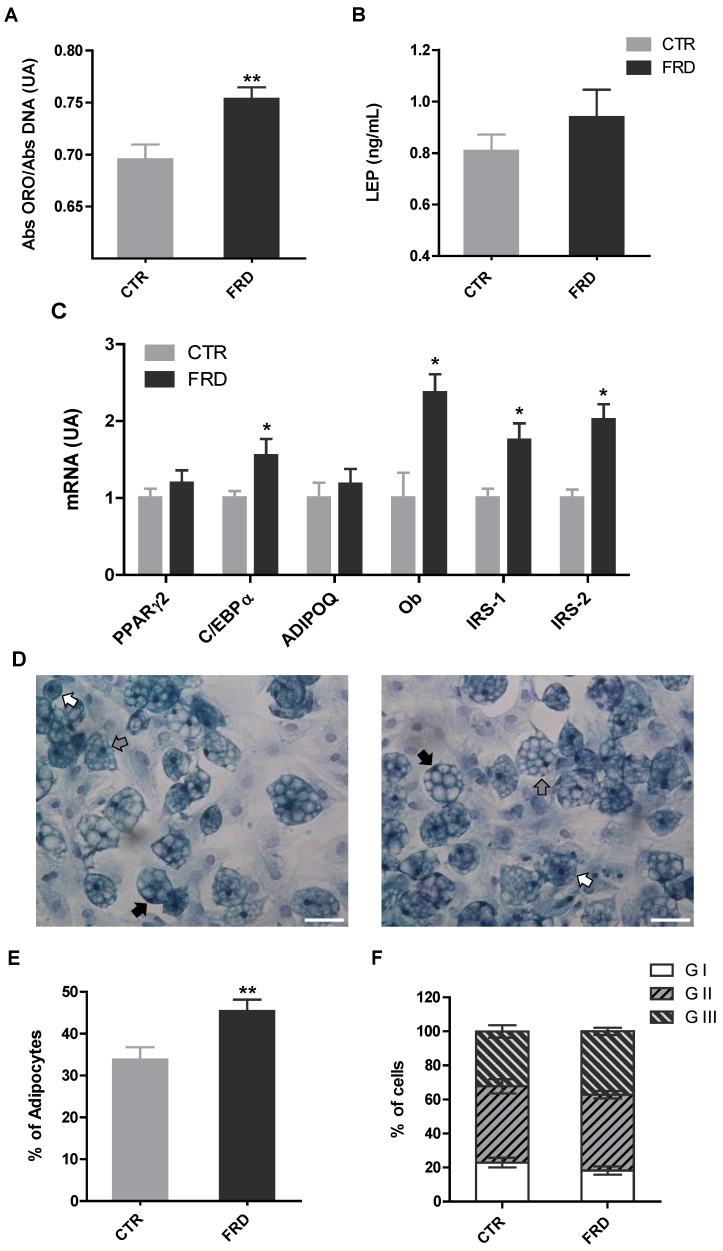
Parameters of terminal adipocyte differentiation. (**A**) Quantification of intracellular lipid accumulation and (**B**) leptin cell secretion (*n* = 5 different experiments with 12 wells per experiment); (**C**) Gene expression of fully-differentiated adipocyte markers (PPAR-γ2, C/EBPα, Adipoq and Ob, IRS-1 and IRS-2) on Dd 10 in cells isolated from CTR and FRD RPAT pads (*n* = 5/6 different experiments; AU: arbitrary units); (**D**) Representative fields containing *in vitro* differentiated CTR and FRD adipocytes (stained on Dd 10, magnification 40×), displaying different degrees of maturation depending on the nucleus position: GI, central (white arrows); GII, displaced from the center (gray arrows); and GIII: fully peripheral (black arrows). Scale bars at 50 μm; (**E**) Percentage of differentiated cells according to the presence of lipid droplets; (**F**) Percentages of cells according to the maturation stage. (*n* = 4/5 different experiments; data from 200/250 cells were recorded 1 in each experiment). Values are means ± SEM. * *p* < 0.05 *vs*. CTR values.

We measured two classical differentiation parameters, such as leptin release and intracellular lipid content in order to determine whether the impact of FRD on APCs adipogenic potential affected, in turn, terminal adipocyte differentiation. We found that while intracellular lipid content increased on Dd 10 (Figure 4A) in FRD cells, leptin release was not modified (Figure 4B). Additionally, the mRNA levels of fully differentiated adipocyte markers indicate that the expression levels of C/EBPα, IRS-1, IRS-2, and Ob genes were higher in FRD than in CTR cells (Figure 4C).

Finally, we evaluated the percentage of differentiated cells and maturation degrees to determine the extent of adipogenesis. Interestingly, we noticed a higher percentage of differentiated adipocytes in RPAT from FRD, whereas the maturation degree in both groups remained the same (Figure 4E,F). These results strongly support that higher APCs competency and number, described above, caused the differentiation increase recorded in FRD adipocytes.

### 3.4. Direct Effect of Fructose Exposure on the Adipogenic Potential of Normal APCs

**Figure 5:**
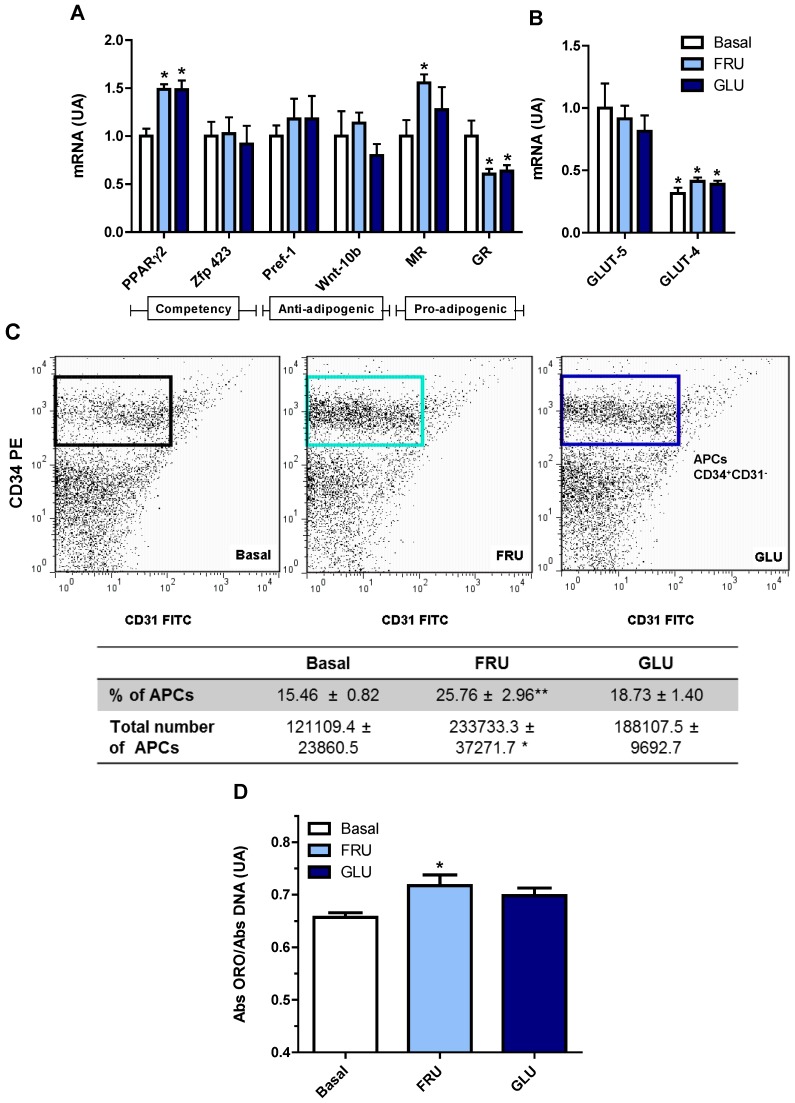
Direct effects of fructose on cultured APCs. (**A**) APCs gene expression of competency (PPAR-γ2 and Zfp423), anti-adipogenic (Pref-1 and Wnt-10b) and pro-adipogenic (GR and MR) markers. * *p* < 0.05 *vs*. CTR values; (**B**) APCs gene expression of specific fructose (GLUT-5) and glucose (GLUT-4) transporters, in Basal and FRU or GLU conditions, in cells from normal rats (AU: arbitrary units). Values are means ± SEM (*n* = 4 different experiments). * *p* < 0.05 *vs*. GLUT-5 values in each group; (**C**) Number of APCs in FRU- and GLU-exposed cells. APCs were identified by FACS analysis using the CD34^+^CD31^−^ profile (boxes). Fluorescence profiles obtained for IgG isotype controls are shown in Figure S2 (Supplementary materials). FITC: fluorescein isothiocyanate; PE: phycoerythrin; (**D**) Intracellular lipid accumulation on Dd10 (*n* = 5 different experiments with 12 wells per experiment). Values are means ± SEM (*n* = 4 different experiments). * *p* < 0.05 *vs*. Basal values.

To examine whether the effect of FRD on normal RPAT APCs was caused, at least in part, by a direct cell exposure to fructose, cells were cultured with FRU and then the APCs adipogenic potential was determined. Our results indicate that normal cells exposed to either FRU or GLU expressed higher levels (*vs*. basal levels) of PPARγ2, without any change in those of Zfp423 (Figure 5A). We also evaluated the mRNA expression level of GLUT-5, which was similarly expressed regardless of the culture conditions, *i.e.*, basal, FRU or GLU (Figure 5B). Similarly to that found in SVF cells (see above), the mRNA expression of GLUT-4 was markedly lower than that of GLUT-5, regardless of FRU or GLU condition. Again, APCs GLUT-4 mRNA levels were similar in all incubation conditions studied (Figure 5B). Interestingly, MR expression level was higher only in FRU-exposed cells, whereas GR expression levels were lower in both FRU- and GLU-exposed cells (Figure 5B).

Additionally we quantified the number of APCs after either FRU or GLU condition. Interestingly, we found that APCs number increased in FRU- but not in GLU-exposed cells (Figure 5C), similarly to what we observed in APCs from FRD-fed rats. It is important to highlight that after seven days of culture, the presence of immune cells was undetectable (Figure S2, Supplementary Materials), and for that reason CD45 was not used in the FACS analysis. Finally, in order to assess whether these APCs changes might increase adipocyte differentiation, we analyzed the *in vitro* intracellular lipid accumulation on Dd 10. We found that FRU-exposed, but not GLU-exposed, cells showed a significantly higher lipid accumulation than that found in cells cultured in basal condition (Figure 5D). Taken together, these results strongly suggest that a direct effect of fructose on the adipogenic potential of APCs might be responsible, at least in part, for the subsequent increase in adipocyte differentiation.

## 4. Discussion

High-fructose feeding has been widely used in animal models to induce similar dysfunctions to those seen in human obesity and metabolic syndrome phenotypes. We previously used FRD intake in rats (10% p/v fructose in drinking water for three weeks) and found numerous metabolic disorders, including peripheral insulin resistance, AAT adipocyte hypertrophy and high oxidative stress, and others [14,15,26]. In the present study the rats were fed a FRD for eight weeks, which was only hyper-caloric during the last five weeks of the experiment. The switch from iso- to hyper-caloric intake could be the result of modifications in the peripheral hormone levels (e.g., ghrelin, leptin and peptide YY (PYY)) and hypothalamic factors (e.g., neuropeptide Y (NPY), pro-opiomelanocortin (POMC)) involved in appetite control, leading to an increase in the caloric intake. It has been suggested that the time required for the establishment of these changes in appetite regulation depends on the amount of fructose ingested [27,28]. Similarly to our previous studies [14,15,26], eight-week FRD intake induced several alterations, including increased plasma levels of INS, LEP and Tg.

The impact of FRD on the development of abdominal obesity has been studied in rodents and humans [15,29]. We have now observed that eight-week FRD intake contributes to abdominal obesity by increasing the RPAT, one of the most representatives AT depots in the rat abdominal cavity. AT depot remodeling after fructose feeding has been reported in a previous study describing larger abdominal adipocytes in contrast to smaller subcutaneous ones [30]. Our results agree with those findings; indeed, adipocyte size analyses from RPAT showed that FRD adipocytes were hypertrophic compared to CTR adipocytes. Besides, adipocyte size distribution showed two adipocyte populations in RPAT from FRD rats: one similar and other larger than CTR adipocyte population, respectively, thus suggesting that RPAT mass expansion in FRD rats may occur from both the combination of newly generated adipocytes through adipogenesis and the hypertrophy of existing adipocytes, respectively.

It is accepted that adipogenesis involves two sequential steps: commitment of mesenchymal stem cells (MSCs) into APCs, acquiring the adipogenic potential and restricting them to the adipocyte linage, followed by terminal adipocyte differentiation [13]. In the first step APCs begin to express CD34, a cell surface antigen that distinguishes between adipogenic and non-adipogenic cell subpopulations [31]. This CD34^+^ cell subpopulation expresses almost exclusively the transcriptional factor Zfp423 [32], which in turn activates the basal expression of PPAR-γ2, a key pro-adipogenic signal that assures APCs conversion into adipocytes [33]. The differential expression of both transcriptional factors determines the competency of APCs, in other words, a cell’s ability to differentiate into adipocytes upon the action of adipogenic stimulus [13]. Finally, in response to the adipogenic stimulus, APCs differentiate into mature adipocytes, cells mainly characterized by intracellular lipid storage and insulin responsiveness.

We were able to assess how the eight-week FRD intake affects competency of APCs from RPAT pads. For this purpose we evaluated the mRNA expression levels of PPARγ2 and Zfp423 in freshly isolated SVF cells. We found that APCs from FRD rats showed an increase in both competency markers, which have also been found after shorter periods of high fructose intake [15], indicating that high APCs competency is maintained between three and eight weeks of FRD intake. This phenomenon might be responsible for the small new adipocyte population observed in FRD rats, as discussed above. FRD intake also induced an increase in APCs MR gene expression without any change in that of GR, both receptors being natural mediators of the pro-adipogenic effects of glucocorticoids [34,35]. However, FRD intake did not modify APCs expression of two anti-adipogenic factors Pref-1 and Wnt10b, thus suggesting that the main FRD effect could be due to the change in APCs competency.

The cell expression pattern of both competency and pro-adipogenic factors in FRD-fed rats reveals an increased APC ability to differentiate into mature adipocytes. Indeed, FRD differentiated adipocytes also showed higher lipid intracellular content, differentiation percentage and expression levels of C/EBPα, Ob and IRS-1/-2 than CTR adipocytes. Collectively, these results indicate that APCs from FRD rats have a greater ability to become mature adipocytes. It is reasonable to speculate that some of the alterations observed in FRD APCs could be due to, in some degree , a direct fructose effect on these cells; in fact, after intestinal absorption a percentage of fructose enters into the systemic circulation and is metabolized by extrahepatic tissues, such as the AT [36]. Both 3T3-L1 preadipocytes and adipocytes express the specific fructose transporter GLUT-5 [17,37]. It has been described that GLUT-5 gene expression is higher in undifferentiated than differentiated 3T3-L1 cells [17], in which it is practically undetectable [17,38], thus indicating that adipocyte precursors are a better target for fructose action than mature adipocytes. Our results showed that cell GLUT-5 expression was not modified by either FRD intake (*in vivo* experiments) or direct fructose treatment (*in vitro* experiments). These results correspond with other reports describing that GLUT-5 is not modulated by an increase in substrate supply [39,40]. However, other authors have shown the opposite [18]. On the other hand, as previously reported [17], we found that GLUT-5 gene expression in both fresh SVF cells and cultured APCs, was greater than that of GLUT-4. Further studies are needed to evaluate any effect on other glucose transporters, such as GLUT-10 and GLUT-12, which are also expressed in adipocytes and SVF cells [41]. There are few available reports regarding direct fructose effects on adipogenesis that have only focused on the terminal differentiation stage of 3T3-L1 preadipocytes [17]. The presence of fructose (55–5500 µM) during adipocyte differentiation induced an increase in PPARγ2, C/EBPα among other pro-adipogenic factors, and either GLUT-5 knockdown or overexpression reduced or increased this effect, respectively [17]. GLUT-5−/− mice have diminished EAT mass compared with wild-type mice, and mouse embryonic fibroblasts derived from GLUT-5−/− mice exhibited impaired adipocyte differentiation [17]. Also, fructose in the culture medium increased lipolysis and the activity of 11-β hydroxysteroid dehydrogenase-1 in differentiated 3T3-L1 cells [18]. To our knowledge, the present work is the first that evaluates direct fructose effects on the early stages of adipogenesis; *i.e.*, before inducing cell differentiation. To this purpose, APCs were grown in presence of FRU for seven days. In this condition APCs expressed higher mRNA levels of PPARγ2 without any change in Zfp423, indicating a greater APCs competency. A similar result was found when cells were cultured with a comparable concentration of GLU. As found in the APCs from FRD rats, FRU did not modify the gene expression of Pref-1 and WNT10b. It has been proposed that the balance between MR/GR plays a key role in the pro-adipogenic effects of glucocorticoids. We found that the APCs grown in the presence of FRU had higher MR and lower GR mRNA levels. Interestingly, no GLU effect on cell MR expression was found. In agreement with this data, it has been described that fructose decreases GR gene expression in adipocytes [42]. Interestingly, when the FRU-exposed APCs were differentiated they accumulated high lipid content. Taken together, these *in vitro* results are quite similar to those we observed in freshly isolated SVF cells from FRD rats.

Another factor that can influence the adipogenic potential in each AT depot is its APCs number. It is generally accepted that subcutaneous AT contains more APCs than AAT [43]. High fat diet intake increases the number of APCs in different AT depots [43,44]. Interestingly, we have previously reported that three-week FRD intake does not induce any change in the number of APCs in RPAT [15]. However, in the present study we evaluated whether a longer period of FRD intake (eight weeks) might alter RPAT APCs number, quantified by using the CD34^+^CD31^−^CD45^−^ FACS pattern [15]. Our results showed an increase in the number of APCs present in RPAT SVF cells from FRD rats. This fact, together with their higher APC competency, clearly indicates a greater differentiation capacity of the RPAT from FRD rats. Additionally, we evaluated the direct effect of fructose on APCs number. Our results showed that FRU directly induced an increase in both CD34^+^CD31^−^ cell number and percentage. However, these results were not observed by using GLU, thus indicating a fructose-specific effect.

## 5. Conclusions

The present study demonstrates that long-term FRD intake altered the RPAT APCs by enhancing their adipogenic potential. Indeed, eight weeks of FRD intake were able to modulate APCs competency and number. Although we previously reported that three weeks of FRD intake modified APCs competency [15], the effect of FRD on APCs number is a novel observation. Moreover and importantly, it has now been assessed that FRU directly modulates the adipogenic competency and number of APCs. In addition, it is important to highlight that the fructose-induced changes in the number/competency of the APCs were not observed with glucose, which agree with the highest GLUT-5 compared with GLUT-4 expression levels in the APCs. It is more than likely that, after both direct and indirect fructose effects, APCs are more capable of generating new adipocytes, which subsequently and as a result of the positive energy balance, will become hypertrophic and contribute to an unhealthy AT mass expansion.

In conclusion, although we demonstrated that fructose intake can activate the adipogenic process in the RPAT, overall the activation of the hyperplasia would not be enough to counteract the hypertrophy. Therefore, predominant hypertrophic RPAT expansion could lead to the development of adiposity dysfunctions, and consequently to the endocrine-metabolic alterations also seen in the human metabolic syndrome/obese phenotype.

## Figures and Tables

**Table 1:** Rat specific primers for qRT-PCR.

	Primers (5′-3′)	GBAN	bp
ACTβ	se, AGCCATGTACGTAGCCATCC	NM_031144	115
	as, ACCCTCATAGATGGGCACAG		
ADIPOQ	se, AATCCTGCCCAGTCATGAAG	NM_144744	159
	as, TCTCCAGGAGTGCCATCTCT		
C/EBPα	se, CTGCGAGCACGAGACGTCTATAG	NM_012524	159
	as, TCCCGGGTAGTCAAAGTCACC		
GLUT-4	se, GCTTCTGTTGCCCTTCTGTC	NM_012751.1	166
	as, TGGACGCTCTCTTTCCAACT		
GLUT-5	se, CTTGCAGAGCAACGATGGAG	NM_031741.1	145
	as, AACTCTGAGGGCGAGTTGAC		
GR	se, TGCCCAGCATGCCGCTATCG	NW_047512	170
	as, GGGGTGAGCTGTGGTAATGCTGC		
IRS-1	se, TGTGCCAAGCAACAAGAAAG	NM_012969.1	176
	as, ACGGTTTCAGAGCAGAGGAA		
IRS-2	se, CTACCCACTGAGCCCAAGAG	NM_001168633.1	151
	as, CCAGGGATGAAGCAGGACTA		
MR	se, TCGCTCCGACCAAGGAGCCA	NM_013131	193
	as, TTCGCTGCCAGGCGGTTGAG		
Ob	se, GAGACCTCCTCCATCTGCTG	NM_013076	192
	as, CTCAGCATTCAGGGCTAAGG		
PPAR-γ2	se, AGGGGCCTGGACCTCTGCTG	NW_047696	185
	as, TCCGAAGTTGGTGGGCCAGA		
Pref-1	se, TGCTCCTGCTGGCTTTCGGC	NM_053744	113
	as, CCAGCCAGGCTCACACCTGC		
Wnt-10b	se, AGGGGCTGCACATCGCCGTTC	NW_047784	175
	as, ACTGCGTGCATGACACCAGCAG		
Zfp423	se, CCGCGATCGGTGAAAGTTG	NM_053583.2	121
	as, CACGGCTGGATTTCCGATCA		

Primers sequences are listed in alphabetical order (se: sense; as: anti-sense; GBAN: GenBank Accession Number; bp: amplicon length in bp).

**Table 2:** Metabolic parameters in CTR and FRD rats.

	CTR	FRD
Body Weight (g)	403.87 ± 10.54	408.69 ± 10.95
LEP (ng/mL)	5.34 ± 0.68	9.39 ± 1.66 *
CORT (nM)	345.21 ± 32.32	282.67 ± 23.36
INS (nM)	0.29 ± 0.04	0.46 ± 0.03 *
Glu (mM)	6.94 ± 0.11	7.05 ± 0.22
Tg (mM)	1.29 ± 0.09	2.51 ± 0.16 *
RPAT mass (g)	3.92 ± 0.29	5.30 ± 0.51 *
RPAT Adipocyte diameter (µm)	69.24 ± 0.67	77.36 ± 0.77 *

Body weight, plasma levels of several metabolites, AT mass and adipocyte size from RPAT in CTR and FRD rats. Values are means ± SEM (*n* = 8 rats per group). * *p* < 0.05 *vs*. CTR values.

## Supplementary Files

Supplementary File 1
