# Supplementary material for: Long-Term Fructose Intake Increases Adipogenic Potential: Evidence of Direct Effects of Fructose on Adipocyte Precursor Cells
Source: Nutrients. 2016 Apr 2;8(4):198. doi: 10.3390/nu8040198 (PMC4848667; doi:10.3390/nu8040198)
Supplement: Supplementary File 1 [file nutrients-08-00198-s001.docx]

Supplementary Materials: Long-Term Fructose Intake Increases Adipogenic Potential: Evidence of Direct Effects of Fructose on Adipocyte Precursor Cells

María Guillermina Zubiría, Ana Alzamendi, Griselda Moreno, María Amanda Rey,
Eduardo Spinedi and Andrés Giovambattista

**Figure S1.** APCs in freshly isolated SVF were identified by FACS analysis using CD34^+^CD45^−^CD31^−^ profile. (**A**) Characteristic dot plot of freshly isolated SVF cells from RPAT without immunostaining. Fluorescence profiles obtained for; (**B**) FITC-conjugated IgG isotype control combined with CD34 PE staining; (**C**) PE-conjugated IgG1 isotype control combined with CD45/CD31 FITC staining. FITC: fluorescein isothiocyanate; PE: phycoerythrin. Values are means ± SEM (*n* = 3/4 different experiments).

**Figure S2.** APCs in cultured SVF cells were identified by FACS analysis using CD34^+^CD31^−^ profile. (**A**) Characteristic dot plot of cultured SVF cells from RPAT without immunostaining. Fluorescence profiles obtained for; (**B**) IgG1 isotype control PE conjugated combined with CD31 FITC staining used to define APCs (black box); (**C**) IgG isotype control FITC conjugated combined with CD34 PE staining. (**D**) CD45 FITC conjugated combined with CD34 PE staining. Red box indicates the CD45^+^ cells. FITC: fluorescein isothiocyanate; PE: phycoerythrin. Values are means ± SEM (*n* = 3/4 different experiments).
